# Temporal instability of the post-surgical maxillary sinus microbiota

**DOI:** 10.1186/s12879-018-3272-9

**Published:** 2018-08-30

**Authors:** Ioannis Koutsourelakis, Ashleigh Halderman, Syed Khalil, Lauren E. Hittle, Emmanuel F. Mongodin, Andrew P. Lane

**Affiliations:** 1 Department of Otolaryngology – Head and Neck Surgery, Johns Hopkins University School of Medicine, Johns Hopkins Outpatient Center, 6th floor, 601 N. Caroline Street, Baltimore, MD 21287-0910 USA; 2 Institute for Genome Sciences, Department of Microbiology & Immunology, University of Maryland School of Medicine, Baltimore, USA

**Keywords:** Post-antrostomy sinus, Stability index, *Staphylococcus*

## Abstract

**Background:**

Chronic rhinosinusitis is an inflammatory disorder in which the role of bacteria remains uncertain. While sinus outflow obstruction is often an initiating event, mucosal inflammation and dysbiosis may persist or develop in sinuses with widely patent surgical openings. Understanding of the relationship between dysbiosis and chronic sinus inflammation is obfuscated by inter-individual microbiota variability and likely intra-individual temporal variation that has yet to be defined. In this study, long-term microbiota stability is investigated within surgically-opened maxillary sinuses of individuals with and without sinus inflammatory disease.

**Methods:**

Maxillary sinus swabs were performed in 35 subjects with longstanding maxillary antrostomies. Subjects with and without active chronic maxillary sinusitis were included. Repeat swabs were obtained from the same sinuses after a prolonged interval (mean 719 ± 383 days). Patients were categorized based on the inflammatory status of the sinus mucosa at times of sample collection, as assessed by nasal endoscopy. Total DNA from swab eluents was extracted, and the microbiota characterized using 16S rRNA gene sequencing followed by taxonomic classification. Prevalence and abundance of genera were determined by analysis of 16S rRNA gene sequences. Taxa were identified that were stably present between two time points in individual subjects.

**Results:**

The overall proportion of stable taxa across time points was 24.5 ± 10.6%. This stability index was consistent across patient groups and not correlated with clinical parameters. Highly prevalent taxa, including *Staphylococcus*, *Corynebacterium*, *Propionibacterium*, and *Pseudomonas*, were often stably present, but varied in relative abundance. *Janthinobacterium*, *Enterobacter*, *Lactobacillus*, and *Acinetobacter* were prevalent and moderately abundant taxa in healthy sinuses, but not in inflamed sinuses. *Moraxella* and *Haemophilus* were present at low prevalence and proportional abundance in chronically or intermittently inflamed sinuses, but not in healthy sinuses.

**Conclusions:**

A relatively small component of the post-antrostomy maxillary sinus microbiota exhibits long-term stability in individual subjects. Stable bacteria include a limited number of highly prevalent and a larger number of lower prevalence taxa, which vary widely in proportional abundance. The concept of individual-specific core sinus microbiota, durable over time and medical therapy, but fluctuating in proportional abundance, has implications for understanding the role of bacteria in CRS pathogenesis.

**Electronic supplementary material:**

The online version of this article (10.1186/s12879-018-3272-9) contains supplementary material, which is available to authorized users.

## Background

Recent interest in the paranasal sinus microbiota, sparked by increased availability of culture-independent methods, has prompted reconsideration of the role of bacteria in chronic rhinosinusitis (CRS). Molecular techniques identify significantly more organisms than standard cultures [1–5], shedding light on complex bacterial communities within healthy sinuses that had previously been assumed to be sterile. The normal microbiota of the sinuses is currently not well defined and appears to vary widely among individuals [2, 6–8]. Evidence suggests that diminished bacterial diversity and richness are associated with CRS [2, 5, 6]. It has been proposed that specific organisms may be critical for sinus health and that some bacterial species may be associated with development of disease [2, 9]. At this time, however, it remains to be elucidated whether a stable sinus microbiome exists, and whether disruption of normal microbial homeostasis underlies alterations in host mucosal integrity, mucociliary clearance, or expression of local inflammatory factors. Also unclear is how an individual’s sinonasal microbiome is established and how dynamic it is over time.

The upper airway serves as a filter, constantly entrapping airborne microbes and particulates in the mucus blanket and removing them harmlessly by mucociliary clearance [10, 11]. In settings of mucostasis, overgrowth can occur of bacteria that are either resident or transiently moving through the sinonasal tract. Acute bacterial sinusitis is a common occurrence that is generally self-limited or responsive to medical treatment, suggesting that acute changes in the sinus microbiome associated with mucostasis are tolerated and reversible. While it is possible that pathological alteration of the microbiota precedes inflammation and subsequent mucostasis, evidence for this is lacking in acute sinusitis. In CRS, a more heterogeneous disorder with a multifactorial etiology [12, 13], purulent static secretions are frequently present, and a role for bacteria in promoting chronic inflammation is generally presumed. Antibiotics are the most common medication prescribed for managing symptomatic CRS, but evidence of efficacy is limited, particularly in CRS with nasal polyposis [14].

In cases of medically recalcitrant CRS, endoscopic sinus surgery (ESS) can be very effective in clearing mucopus and enlarging drainage pathways, in order to facilitate mucociliary function and access for topical therapies [15, 16]. The restoration of sinus health after ESS again indicates that changes in the microbiota associated with chronic inflammation are also caused, or at least perpetuated, primarily by mechanical obstruction. In a subset of patients, however, ESS does not reverse the chronic inflammatory process, requiring long-term medical management and sometimes repeated surgery. The role of bacteria in the pathogenesis of chronic sinus mucosal inflammation in this setting remains under investigation [17]. In patients who have undergone adequate ESS, ongoing sinusitis is not due to blockage of sinus ostial outflow. Often, periods of disease control are punctuated by acute inflammatory and/or infectious exacerbations, which can be readily visualized endoscopically and sampled in the office setting. In this way, post-operative sinus cavities present a window through which to investigate the relationship between the microbiome and the mucosal inflammatory state over time.

Previous studies suggest that the sinus microbiome varies among individuals, among sub-sites within an individual sinonasal tract, and in response to medical therapy [18–20] or sinus surgery [21–23]. While the microbiota remain remarkably constant over time in the gut and urogenital tract [24–26], temporal variation has not been evaluated in the post-operative sinuses. In order to evaluate the hypothesis that dysbiosis is a contributing factor in surgically-recalcitrant CRS, it is important to establish what constitutes healthy microbiota and the degree to which a stable set of bacteria, or “core” sinus microbiome, exists for individuals. Only with this understanding can the microbiota characteristics associated with chronic inflammatory sinus disease be interpreted. Therefore, the aim of this study is to determine the long-term stability of the microbiota within surgically-opened maxillary sinuses between two temporally separate points. We hypothesize that stable microbiota features exist within individuals, despite changes in disease state and medical therapy.

## Methods

This prospective study was approved by the Johns Hopkins Medicine Institutional Review Board. All subjects provided written consent to participate. Between 3/2011 and 4/2015, 35 patients with longstanding patent maxillary antrostomies, status post ESS in the remote past, had swab samples of the maxillary sinus taken at two distant time points as part of a routine follow-up. All collections were taken from the maxillary sinus mucosa using a cotton-tipped wire swab (Polyester Dacron Swab Pack, MPC, Amarillo, CA; Dry Swabs, Copan Diagnostics, Murrieta, CA) under endoscopic visualization by the same clinician (APL). Swabs were discarded and retaken if contaminated by mucosa outside the target region. Immediately following collection, the samples were flash frozen and stored at −80C until processed.

### Definition of groups of patients

The subjects were separated into three groups based on the presence or absence of chronic maxillary sinusitis at the times of sampling, as defined by the EPOS [27] and AAO-HNS guidelines [28] and by endoscopic evidence of inflammation: Group 1 (no CRS): chronic maxillary sinusitis not present at either time point; Group 2 (persistent CRS): persistent chronic maxillary sinusitis at both time points; Group 3 (CRS exacerbation): clinical evidence of chronic maxillary sinusitis exacerbation at one time point only.

In this study design, each subject acts as his/her own control. In other words, one time point can be considered the “baseline” to which the other time point is compared. Additionally, groups 1 and 2, which had the same inflammatory state at both time points, serve as controls for group 3, where different inflammatory states existed at each time point.

The electronic medical records of the patients were reviewed for demographic and clinical information including current sinonasal medications, and recent courses of antibiotics (within 8 weeks of sampling).

### DNA extraction

Total genomic DNA was extracted using a protocol developed at the University of Maryland School of Medicine—Institute for Genome Sciences and previously described [29, 30]. Briefly, samples were thawed on ice, incubated in an enzymatic cocktail containing lysozyme, mutanolysin, proteinase K and lysostaphin, after which the microbial cells were lysed using bead beating with silica beads (Lysing Matrix B, MP Biomedicals) with the FastPrep instrument (MBio, Santa Ana, CA). The DNA was then further extracted and purified using the Zymo Fecal DNA kit (Zymo Research, Irvine, CA).

### 16S rRNA gene PCR amplification and sequencing

The universal primers 319F (ACTCCTACGGGAGGCAGCAG) and 806R (GGACTACHVGGGTWTCTAAT) were used for PCR amplification of the V3-V4 hypervariable regions of the 16S rRNA gene in 96-well microtiter plates using procedures previously published [31]. The 806R primer included a unique 12 base pair (bp) sequence tag to barcode each sample, so that up to 500 samples could be multiplexed in a single Illumina MiSeq (Illumina, San Diego, CA) run. PCR amplifications were performed using Phusion High-Fidelity DNA polymerase (Thermo Fisher Scientific, Waltham, MA) and 10 ng of template DNA in a total reaction volume of 25 μl. Reactions were run in a DNA Engine Tetrad 2 thermo cycler (Bio-Rad, Hercules, CA) using the following cycling parameters: 30 s at 98 °C, followed by 30 cycles of 10 s at 98 °C, 15 s at 66 °C, and 15 s at 72 °C, with a final step of 10 min at 72 °C. The number of PCR cycles chosen is within the range used for standard PCR in molecular evolution and environmental studies [32]. Negative controls without a template were processed for each primer pair. The presence of amplicons was confirmed using gel electrophoresis, after which the SequalPrep Normalization Plate kit (Thermo Fisher Scientific, Waltham, MA) was used for clean-up and normalization (25 ng of 16S PCR amplicon was pooled for each sample), before pooling and 16S rRNA sequencing using the Illumina MiSeq (Illumina, San Diego, CA) (according to the manufacturer’s protocol).

### Sequence quality filtering and analysis of 16S rRNA gene sequences

16S rRNA reads were initially screened for low quality bases and short read lengths [33]. We used criteria previously described to assess the quality of sequence reads [34]. Briefly, to pass, a sequence read had to (a) include a perfect match to the sequence tag (barcode) and the 16S rRNA gene primer; (b) be at least 200 bp in length; (c) have no undetermined bases; and (d) have at least a 60% match to a previously determined 16S rRNA gene sequence. Paired-end read pairs were then assembled using PANDAseq [35] and the resulting consensus sequences were de-multiplexed (i.e. assigned to their original sample), trimmed of artificial barcodes and primers, and assessed for chimeras using UCHIME [36] in de novo mode implemented in Quantitative Insights Into Microbial Ecology (QIIME release v. 1.9) [37]. Quality trimmed sequences were then clustered de-novo into Operational Taxonomic Units (OTUs) at 97% similarity cutoff using the uclust algorithm in QIIME, and taxonomic assignments were performed using the Ribosomal Database Project (RDP) classifier implemented in QIIME and the Greengenes database (release 13.8) as a a reference. Singleton sequences were then removed. Estimating and visualization of alpha-diversity indices was performed using R statistical software v.3.2 (Core Team 2013) with packages ggplot218 and Phyloseq v. 1.12.219 [38, 39]. Because alpha diversity metrics can be susceptible to uneven sampling depth between samples, alpha diversity measures were compared after rarefaction to the minimum sampling depth of 500 sequences. When appropriate, taxonomic assignment data were normalized to account for uneven sampling depth with metagenomeSeq’s cumulative sum scaling (CSS; implemented in R) [40].

### Statistical analysis

Data were analyzed using SPSS 23.0 (SPSS INC, Chicago, IL). Quantitative data are reported as mean ± SD. The normality of the data distributions was assessed by the Kolmogorov-Smirnov test. One-way ANOVA was used for multiple comparisons between groups, followed by the Scheffé test for post hoc analyses, as appropriate. Linear regression analysis was performed using the least squares method in order to investigate the relationship between different variables. In order to study each individual’s microbiota over time, paired samples from an individual were assessed, and the fraction of shared genera between them was determined, as measured by the binary Jaccard index [41]. For instance, we calculated the fraction of shared genera within a subject between two samples collected hundreds of days apart [Jaccard index (Sample A, Sample B) = SampleA∩SampleB/SampleA∪SampleB]. Microbiota biodiversity metrics included observed OTUs, Shannon diversity index, average proportional abundance defined as the proportional presence of each taxa in a sample, and prevalence defined as the presence or absence of each taxa in a sample. Beta diversity comparisons were performed from CSS-normalized data through principal coordinates analysis plots of Bray-Curtis dissimilarity index determined using QIIME and tested for significance with analysis of similarity (ANOSIM) algorithm (9999 permutations) [42] implemented in the Vegan package in R. A *p* value of < 0.05 was considered to indicate statistical significance.

## Results

### Patient demographics

All 35 patients (8 male, 27 female) enrolled in the study and had maxillary sinus samples collected at widely separated time points as described. The demographic characteristic of the whole study group and the three groups separately (group 1: no CRS, group 2: persistent CRS, group 3: CRS exacerbation) are summarized in Tables 1 and 2 respectively. As can be seen, the time interval between sampling was 719 ± 383 days [median (interquartile range) 679 days (490–917 days)]. There were no significant differences in gender, age, or time elapsed between sample collections in the three groups. Groups 2 and 3 had significantly more subjects with nasal polyps than group 1. Group 2 subjects had significantly greater use of oral steroids compared to the other groups, and greater use of nasally-irrigated steroids as compared to group 1. Four patients (four women; two from group 2 and two from group 3) received oral or topical antibiotics during the course of the study. The characteristics of the study cohort without these four patients is presented in Tables S1 and S2 of the Additional file 1.

**Table 1:** Patients’ characteristics

Study participants, *n*	35
Age, years	55.0 ± 13.5
Male, *n* (%)	9 (25.7)
Time interval between sampling, days	719 ± 383
Subjects with nasal polyps, %	20 (57.1)
Patients on steroid rinses, %	16 (45.7)
Patients on oral steroid, %	3 (8.6)
Patients on topical antibiotic, %	3 (8.6)
Patients on antibiotics, %	3 (8.6)
Patients on steroid spray, %	11 (31.4)
Patients on saline rinsing, %	31 (88.5)
Patients on antibiotics the past 8 weeks, %	1 (2.8)

Data are presented as mean ± SD

**Table 2:** Patient characteristics, medication use and microbiome data of the three groups

	Group 1: No CRS (*n* = 9)	Group 2: Persistent CRS (*n* = 11)	Group 3: CRS exacerbation (*n* = 15)
Age, years	57.9 ± 13.1	57.4 ± 13.9	51.6 ± 13.5
Male, %	2 (22.2)	3 (33.3)	4 (44.4)
Patients with polyps, %	1 (11.1)	9 (81.8)*	10 (66.7)*
Time intervals, days	747 ± 226	761 ± 414	673 ± 448
Patients on steroid rinses, %	11.1	63.6*	53.3
Patients on oral steroid, %	0	27.2**	0
Patients on topical antibiotic, %	0	18.1	6.7
Patients on antibiotics, %	11.1	0	13.3
Patients on steroid spray, %	22.2	45.5	26.7
Patients on saline rinsing, %	77.8	100	86.7
Patients on antibiotics the past 8 weeks, %	0	9.1	0
Shannon diversity index	2.08 ± 1.18	1.52 ± 0.67**	1.92 ± 0.81
Jaccard index	24.2 ± 10.7	25.3 ± 12.7	23.6 ± 11.3

Data are shown as mean ± standard deviation unless otherwise specified. *: *p* < 0.05 versus group 1; **: *p* < 0.05 versus group 1 and 3

### Maxillary sinus microbiome

**Fig. 1:**
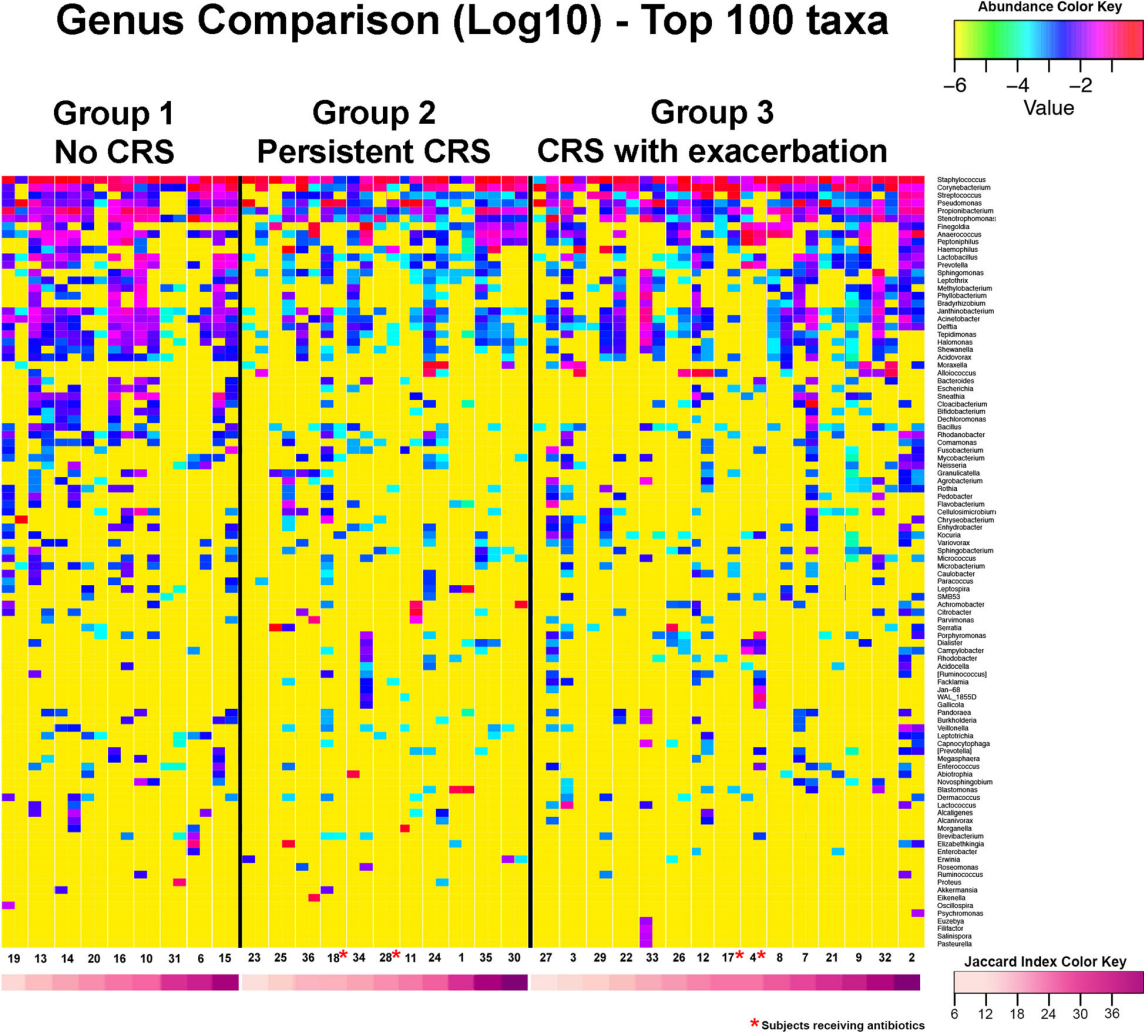
Microbiota of the post-operative maxillary sinus in repeated samples from 35 subjects (patients receiving antibiotics within 8 weeks are noted with red asterisk). In this heatmap visualization, the subjects are divided into groups based on the presence or absence of maxillary sinusitis at the time of sample collection. Each column represents one sample, and subject numbers are listed under paired samples from the same individual. Each row is a bacterial taxon, with the proportional abundance of that taxon depicted by color corresponding to the annotated key (*top right*). Last row represents Jaccard index of each sample which is depicted by color corresponding to the annotated key (*bottom right*). The stability index remained similar across groups, suggesting that disease state does not significantly affect this β-diversity metric

The 16SrRNA gene sequencing data indicated a total of 314 bacterial genera were detected in the maxillary sinuses of the study group (Additional file 2). Figure 1 shows a heat map representation of the taxa identified in all samples from the three group of patients as well as the Jaccard index of each sample. Across all samples analyzed, *Staphylococcus* was highly prevalent, identified in 100% of samples, while Pseudomonas was found in 82.7 of samples and *Propionibacterium* and *Corynebacterium* were each present in 76.9% of samples. *Staphylococcus*, *Corynebacterium*, and *Haemophilus* were the most abundant genera in post-antrostomy maxillary sinuses. The most prevalent and abundant genera are shown in Tables 3 and 4.

**Table 3:** Fifteen most prevalent (*left*) and proportionally abundant (*right*) genera in the post-antrostomy maxillary sinus

Genus	Prevalence (%)
1. *Staphylococcus*	100
2. *Pseudomonas*	82.7
3. *Propionibacterium*	79.9
4. *Corynebacterium*	76.9
5. *Stenotrophomonas*	65.6
6. *Streptococcus*	59.9
7. *Anaerococcus*	59.9
8. *Acinetobacter*	45.6
9. *Janthinobacterium*	42.7
10. *Lactobacillus*	39.9
11. *Enterobacter*	37.1
12. *Streptophyta*	36.2
13. *Delftia*	36.1
14. *Sphingomonas*	33.2
15. *Tepidimonas*	33.2

*Staphylococcus* is stably prevalent and abundant, while the proportional abundance of the other highly prevalent taxa, including *Pseudomonas*, *Propionibacterium*, *Corynebacterium*, *Stenotrophomonas*, *Streptococcus* and *Anaerococcus* is relatively low

**Table 4:** Fifteen most prevalent (*left*) and proportionally abundant (*right*) genera in the post-antrostomy maxillary sinus

Genus	Mean Proportional Abundance (%)
1. *Staphylococcus*	32.6
2. *Corynebacterium*	10.0
3. *Haemophilus*	9.25
4. *Pseudomonas*	5.99
5. *Streptococcus*	4.97
6. *Anaerococcus*	4.64
7. *Proprionibacterium*	3.86
8. *Moraxella*	3.62
9. *Stenotrophomonas*	3.35
10. *Enterobacter*	3.30
11. *Serratia*	2.39
12. *Finegoldia*	2.10
13. *Alloiococcus*	1.68
14. *Neisseriaceae*	1.65
15. *Methylobacteriaceae*	1.62

*Staphylococcus* is stably prevalent and abundant, while the proportional abundance of the other highly prevalent taxa, including *Pseudomonas*, *Propionibacterium*, *Corynebacterium*, *Stenotrophomonas*, *Streptococcus* and *Anaerococcus* is relatively low

**Fig. 2:**
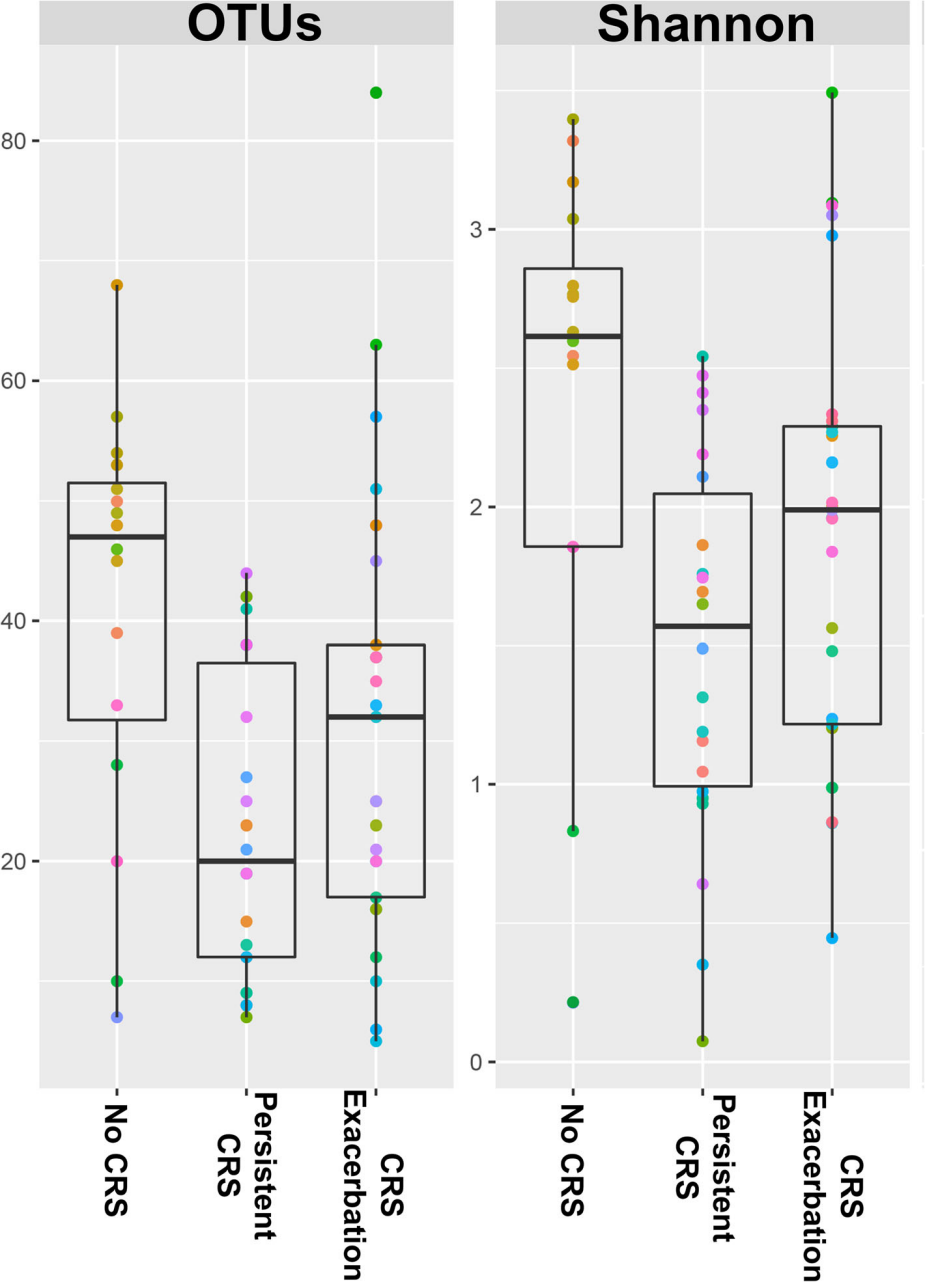
Box and whisker plots comparing observed OTUs and diversity (Shannon diversity index) in the three groups of patients. The “box” represents the interquartile range, the thick horizontal bar shows the median value and the “whiskers” represent the minimum and maximum values. Each color represents the paired samples from an individual subject. OTUs: operational taxonomic units; #: *p* < 0.05

For all subjects, the Shannon diversity index was 1.83 ± 0.88. Figure 2 shows observed OTUs and Shannon diversity index in the three groups. Patients of group 2 had significantly lower richness (as expressed by observed OTUs) and diversity compared to the other two groups (Table 2).

### Stability of the microbiome over time

**Fig. 3:**
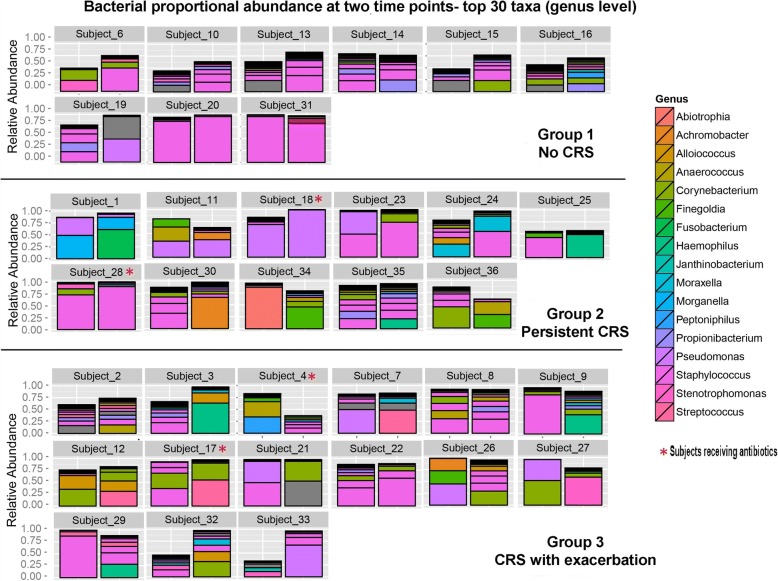
Relative abundance of top 30 taxa found in repeated samples from the same individual. Group 1: no maxillary sinusitis at either time point; Group 2: persistent maxillary sinusitis at both time points; Group 3: CRS exacerbation at one time point only. In the bar chart, the proportional abundance is denoted by the size of the bar segment, and the identity of the bacterial taxon is denoted by the color, as defined in the key at right

**Fig. 4:**
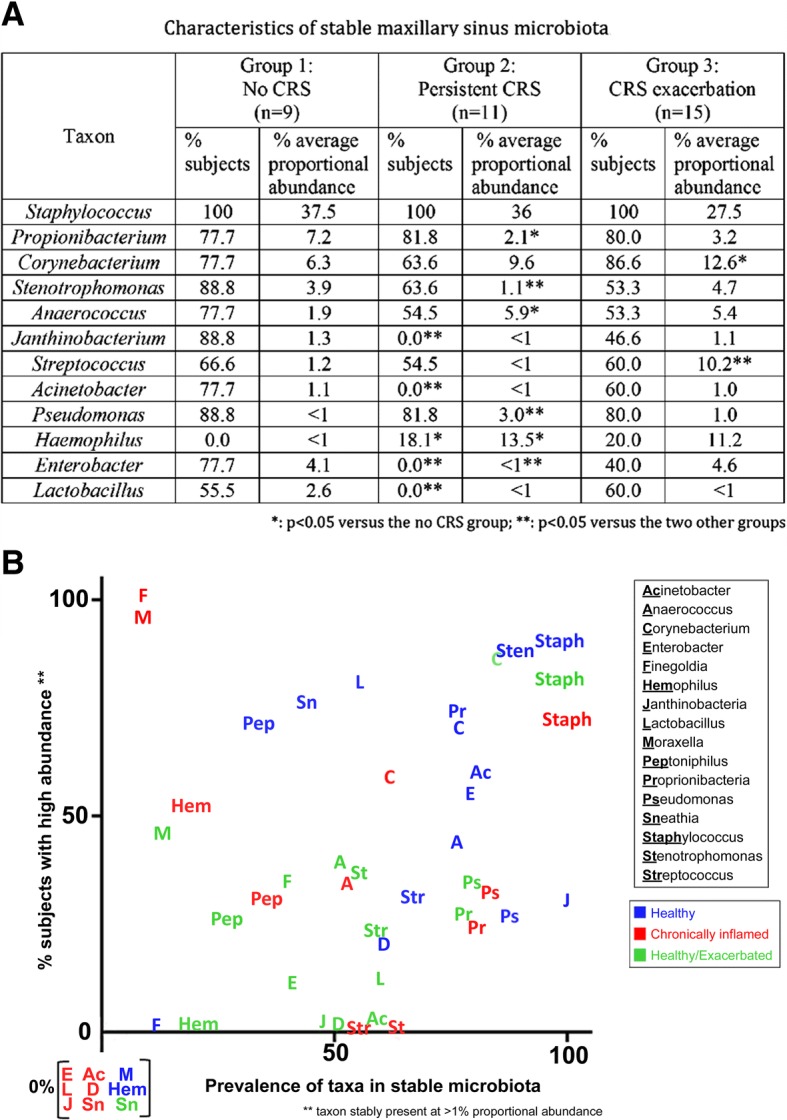
**a** Characteristics of the stable post-antrostomy maxillary sinus; **b** Graph depicting the relationship between the prevalence of stable taxa and their level of proportional abundance when present. The X-axis shows the prevalence of the taxa at any abundance, and the Y-axis shows the percentage of those subjects in whom the proportional abundance is greater than 1%. Taxa in the upper right are highly prevalent at a high abundance, while taxa in the bottom right are highly prevalent but at relatively low abundance

In Fig. 3, the microbiota of paired samples are compared, limited to the top 30 genera. The stability of the bacteria present in paired samples from subjects was assessed using the Jaccard index. This demonstrated that an average of 24.5 ± 10.6% of maxillary sinus genera present at one time point are also present at the other. As shown in Table 2, Jaccard index didn’t differ between the three groups (24.2 ± 10.7 for group 1; 25.3 ± 12.7 for group 2; 23.6 ± 11.3 for group 3) Fig. 4 shows the characteristics of the stable maxillary sinus microbiota in the three groups of patients (i.e. taxa present at both time points). Among the highest prevalence organisms, *Staphylococcus* is stably abundant in all groups, while the proportional abundance of the other highly prevalent taxa, including *Propionibacterium*, *Corynebacterium*, *Stenotrophomonas*, *Streptococcus*, and *Pseudomonas*, varies significantly among groups. Several organisms, including *Campylobacter* and *Sphingomonas*, are highly prevalent in individual subjects at one time point or the other, but rarely present stably at both times. In healthy sinuses, *Janthinobacterium*, *Enterobacter*, *Lactobacillus*, and *Acinetobacter* are prevalent and moderately abundant taxa, while these organisms are absent or much less prevalent in inflamed sinuses (Fig. 4). Other bacteria, including Moraxella and Haemophilus, were never found in the stable microbiota of healthy subjects, but were present at low prevalence and proportional abundance in subjects with chronic or intermittent inflammation (Fig. 4).

**Fig. 5:**
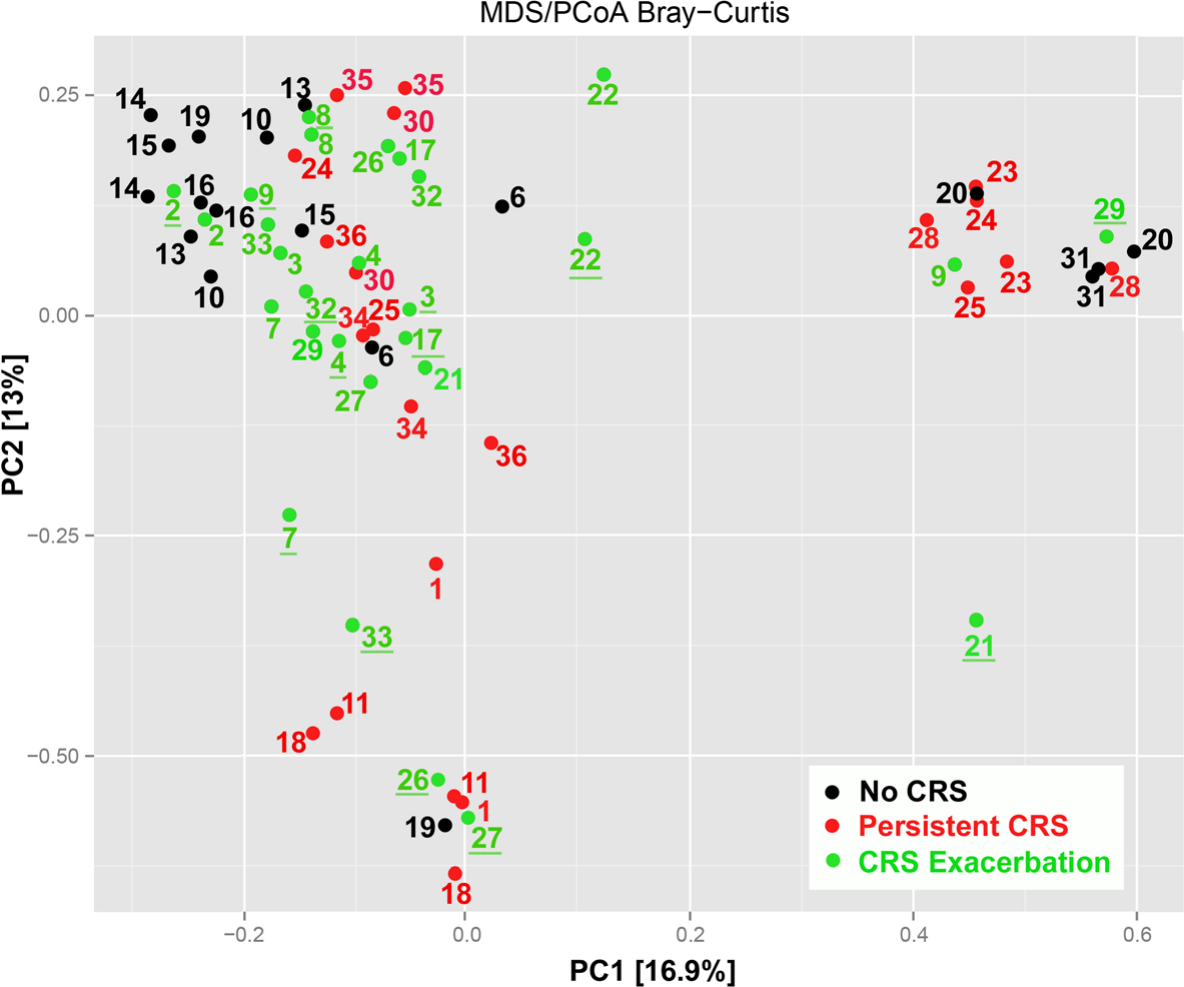
Principal coordinate analysis of Bray–Curtis dissimilarity index generated from taxa of the three groups at both time points. Proportion of variance explained by each principal coordinate axis is denoted in the corresponding axis label. Colored by group and tested with ANOSIM (*R* = 0.28, *p* = 0.001). Black circles: group 1; red circles: group 2; green circles: group 3. Inflamed time points for group 3 are underlined

Beta-diversity of nasal microbiota was visualized using ordination. Bray-Curtis dissimilarity index that takes into account the abundance of each OTU (i.e. structure of the microbiome) and estimates of the dissimilarity of the samples was used. ANOSIM tested the hypothesis that samples cluster more closely within patients, rather than between groups. Contrary to our hypothesis, within-patient sample pairs did not cluster together nearly as strongly as they did by group (ANOSIM *R* = 0.28, *p* = 0.001; Fig. 5). Figure 5 presents the principal coordinates analysis, where the inflamed time points for group 3 are underlined.

### Association of stability with clinical parameters

Linear regression analysis did not demonstrate any association between stability index and gender, age, disease state, saline irrigation, topical or systemic steroids, or antibiotics (Table 5).

**Table 5:** Simple linear regression analysis models for Jaccard index in all patients

Independent variable	*B*	SE	*r* ^2^	*P-*value
Age, years	0.213	0.131	0.074	0.114
Gender (1 = male, 2 = female)	0.943	4.153	0.002	0.822
Polyps (1 = yes, 2 = no)	0.659	3.669	0.001	0.859
Saline irrigation	−4.089	6.556	0.012	0.537
Topical steroids	4.980	3.913	0.053	0.213
Systemic steroids	1.616	6.500	0.002	0.805
Antibiotics	3.731	6.469	0.011	0.569
Group (1 = group 1, 2 = group 2, 3 = group 3)	−0.272	1.459	0.001	0.853

*B*: parameter estimate; *SE* standard error

## Discussion

The vast majority of patients with CRS experience clinical resolution after medical and surgical therapy, suggesting that sinus dysbiosis, if present, is either fully reversible or is secondary to impaired ventilation and mucociliary function. In cases of persistent CRS after surgery, the ongoing role of bacteria is unknown. The nose and sinuses are not sterile, and therefore bacteria are necessarily found in both healthy and CRS maxillary sinuses after antrostomy. In this study, we compared the maxillary sinus microbiota in a cohort of patients from whom samples were obtained years apart. Our results confirm that the post-operative maxillary sinus (POMS) microbiota is broadly characterized by a group of highly prevalent taxa which differ widely in proportional abundance. Additionally, there is a diverse sinus microbiota that contains an assortment of proportionally low abundance bacteria that vary between individuals and within individuals over time. The POMS microbiota of an individual person is relatively unstable, with an overlap of approximately 25% of taxa between samples. A slightly higher degree of stability is present in the setting of chronic inflammation where there is a depleted, low diversity microbial community. Additionally, beta-diversity metrics that account for the relative abundance of taxa (Fig. 5), show that sample pairs cluster together more strongly by group than they did within-patient. These findings highlight the complexity of identifying a “normal” or “healthy” microbiome upon which to propose a dysbiotic mechanism responsible for driving CRS. As an individual’s POMS microbiota may be in constant temporal flux, perhaps due to differences in inhaled and skin bacteria transiently entering and being cleared from the sinus, the cause and effect relationship of specific bacteria present in CRS may be difficult to demonstrate. Our findings are in accordance with the results of a recent study, which reported on the middle meatus microbiome of 23 patients undergoing ESS [22]. The authors found a large number of taxa that constitute an extremely small fraction of the total detected microbiota [22].

Previous studies have revealed unique “cores” of bacterial organisms that remains present in samples across various time periods in individual body sites. The gut, which has been studied the most extensively, has a stable core group of bacteria in various proportions. Martinez et al. found 33–40 species-level OTUs that remained consistent over 1 year in three subjects [43]. While this was only 12% of the total OTUs detected, those species accounted for >75% of the total sequences obtained for each subject. Faith et al. studied individual subjects’ fecal microbiota over a 5-year period, showing it to be quite stable with 60% of bacterial strains remaining present [25]. Additionally, the most stable components of the fecal microbiota were also the most abundant. Caporaso et al. [24] extended their study to include the mouth and palms as well as the gut. They too identified a small “core human microbiota” at these body sites present over months, representing about 10% of all OTUs identified at each site [24]. Interestingly, the size of the stable core microbiome was largest for mouth followed by the gut and then the palms respectively. The microbiome of the nasal passages of healthy individuals has been reported to be relatively stable, although a stability index was not calculated [44]. Futhermore, Yan et al. [20] examined the microbiota of the anterior nasal cavity, middle meatus, and sphenoethmoid recess in 12 healthy adults, noting a temporally stable, highly individualized baseline, with some differences in microbiota between the anterior nares and deeper anatomic subsites. Another study that examined middle meatus swabs from 28 healthy subjects found that many individuals harbor respiratory pathogens at low abundance, suggesting that pathogenic bacteria may be transient or permanent members of the healthy microbiome at relatively low amounts [8]. This is consistent with our findings in the POMS.

In the gut, the composition of an individual’s stable microbiota is proposed to be derived from diet or environmental exposures from early in life. In contrast, our findings suggest that the POMS microbiota is much more dynamic. A 25% stability at the genus level may indicate a significant and ongoing influence of transient environmental exposures in the upper airway, which, like the palms, are likely more varied than the gut. At the same time, a number of highly prevalent taxa in the POMS are largely stable in the microbiota but shift in abundance over time. Bacteria belonging to the *Staphylococcus*, *Corynebacterium*, *Proprionibacterium* and *Pseudomonas* genera are the primary examples. These organisms are often among the most abundant taxa found at any given time point, and, when this is the case, they are present at the other time point in over 80% of subjects.

A limitation of this study is that only two points in time are compared. Obviously, collection of more frequent time points over a greater period would most accurately portray maxillary sinus microbiota temporal variation. We performed a preliminary analysis of additional time points in a smaller number of subjects, which suggested increased microbiota stability over short time intervals, but still with marked fluctuation. As more longitudinal samples are collected and analyzed for individual subjects, characterization of the stably present organisms can be refined. It is likely that medications, dietary influences, and changes in living or work environments lead to shifts in sinus microbiota between sampling points. This is expected and important to recognize in a “real-world” descriptive study such as this. The percentage range of the stable taxa is fairly consistent, despite individual differences in multiple external factors. Indeed, even if we excluded patients who were on antibiotics during sampling or have been on antibiotics in the 2 months preceding sampling, the stability index remains unchanged. The precision to which rare taxa can be identified in the specimens may limit interpretation to some extent. However, based on the depth of coverage, the fact that samples are compared within individuals, and that negative controls without a template were processed for each primer pair during PCR, the possibility of contamination has been minimized as much as possible. Accordingly, we believe that rare taxa were accurately detected and their inclusion is justified in the stability analysis. We observe that very rare taxa present at one time point may be present in high relative abundance at a subsequent time point. This finding highlights the dynamic nature of the sinus microbiota and argues that rare taxa should not be automatically dismissed as irrelevant or contaminants in microbiome studies. Lastly, it should be noted that increased PCR cycle numbers are associated with point mutation artifacts and reduced richness, but do not appear to affect community structure [45].

To the extent that “core” post-antrostomy maxillary sinus microbiota exists, it is likely that these bacteria have the capacity to participate in a “healthy” diverse microbiome as well as to pathologically dominate in a disease setting. The reason for the decreased diversity and evenness associated with CRS is not understood, and a cause-and-effect relationship has not been demonstrated. It is conceivable that changes in the local milieu due to chronic inflammation support the growth of certain resident bacterial species over others, thus driving the community to a less diverse state. Alternatively, development of uneven and less diverse microbiota within the sinus may precede and stimulate a subsequent inflammatory response. Our data suggest that clinically healthy POMS are associated with diverse but relatively temporally unstable microbiota. In cases of longstanding infectious CRS without polyps, a highly depleted microbiota may develop, dominated by few stable taxa. This subset of patients can be frustratingly difficult to rid of inflammation and infection, and this disease phenotype seems most consistent with an etiology linked to dysbiosis. Although long-term control of inflammation is also challenging in CRS with nasal polyps, the association with infection or dysbiosis is not apparent, and the present study shows a similar level of temporal stability to healthy sinuses, with a reduced overall diversity.

## Conclusions

Culture independent techniques permit a more comprehensive view of the sinus microbiome than previously available, but interpretation of this information has presented new challenges. Although dysbiosis continues to be hypothesized as contributing to the pathogenesis of CRS, studies to date have not revealed a consistent pattern differentiating CRS patients and healthy controls. In focusing on the post-surgical maxillary sinus, our group has described shifts in the microbiota occurring in response to medical therapy for acute infectious CRS exacerbation, as well as the broad impact of topical therapies [18, 19]. Taken together with the present study of temporal variation, it is clear that the sinus microbiome is complex, variable, and likely influenced constantly by internal and external factors. The identification of temporally stable taxa in uninflamed sinuses may lend further support for their role in maintaining health, but against the larger backdrop, much further investigation is necessary to delineate the relationship between these bacteria and CRS, before consideration can be given to concepts such as probiotic therapy or selection of antibiotics based on culture-independent methods.

## Additional files

Additional file 1: **Table S1.** Patients’ characteristics (excluding patients receiving antibiotics with 8 weeks of sample collection). **Table S2.** Patient characteristics, medication use and microbiome data of the three groups (excluding patients receiving antibiotics with 8 weeks of sample collection). (DOC 39 kb)

Additional file 2: 16S rRNA gene sequences of all samples presented by group. (XLS 255 kb)

## Abbreviations

ANOSIM: Analysis of similarity
bp: base pair
CRS: Chronic rhinosinusitis
CSS: Cumulative sum scaling
ESS: Endoscopic sinus surgery
OTUs: Operational taxonomic units
POMS: Post-operative maxillary sinus
QIIME: Quantitative insights into microbial ecology
RDP: Ribosomal database project
