# Supplementary material for: Temporal instability of the post-surgical maxillary sinus microbiota
Source: BMC Infect Dis. 2018 Aug 30;18:441. doi: 10.1186/s12879-018-3272-9 (PMC6117940; doi:10.1186/s12879-018-3272-9)
Supplement: Supplementary file 1 — Table S1. Patients’ characteristics (excluding patients receiving antibiotics with 8 weeks of sample collection). Table S2. Patient characteristics, medication use and microbiome data of the three groups (excluding patients receiving antibiotics with 8 weeks of sample collection). (DOC 39 kb) [file 12879_2018_3272_MOESM1_ESM.doc]

**Additional file 1**

Table S1. Patients’ characteristics (excluding patients receiving antibiotics with 8 weeks of sample collection).

| Study participants, n | 31 |
| --- | --- |
| Age, years | 55.2±12.4 |
| Male, n (%) | 9 (29.0) |
| Time interval between sampling, days | 739±420 |
| Subjects with nasal polyps, % | 18 (58.0) |
| Patients on steroid rinses, % | 15 (48.3) |
| Patients on oral steroid, % | 2 (6.4) |
| Patients on steroid spray, % | 9 (29.0) |
| Patients on saline rinsing, % | 29 (93.5) |

Data are presented as mean± SD.

Table S2. Patient characteristics, medication use and microbiome data of the 3 groups (excluding patients receiving antibiotics with 8 weeks of sample collection).

|  | Group 1:  No CRS  (n=9) | Group 2:  Persistent CRS  (n=9) | Group 3:  CRS exacerbation (n=13) |
| --- | --- | --- | --- |
| Age, years | 57.9±13.1 | 57.4±13.9 | 51.6±13.5 |
| Male, % | 2 (22.2) | 3 (33.3) | 4 (30.7) |
| Patients with polyps, % | 1 (11.1) | 9 (81.8)* | 10 (66.7)* |
| Time intervals, days | 747±226 | 768±405 | 698±477 |
| Patients on steroid rinses, % | 11.1 | 77.7* | 53.8 |
| Patients on oral steroid, % | 0 | 22.2** | 0 |
| Patients on steroid spray, % | 22.2 | 44.4 | 23.7 |
| Patients on saline rinsing, % | 77.8 | 100 | 92.3 |
| Shannon diversity index | 2.08 ± 1.18 | 1.49±0.72** | 1.88±0.90 |
| Jaccard index | 24.2±10.7 | 25.3±12.7 | 23.6±11.3 |

Data are shown as mean ± standard deviation unless otherwise specified. *: p<0.05 versus group 1; **: p<0.05 versus group 1 and 3.
